# Evaluating Anxiety Levels and Physical Activity Among the Population of Saudi Arabia

**DOI:** 10.3390/medicina61050862

**Published:** 2025-05-08

**Authors:** Anwar A. Sayed, Ghaida Ghassan Alsisi, Amjad Faisal Aljohani, Manal Salman Aloufi, Samiyah Saleh Alhejaili, Reem Mebrek Almatrafi

**Affiliations:** 1 Department of Basic Medical Sciences, College of Medicine, Taibah University, Medina 42353, Saudi Arabia; 2 College of Medicine, Taibah University, Medina 42353, Saudi Arabia

**Keywords:** anxiety, GAD-7, general anxiety disorder, GPAQ, physical activity, Saudi Arabia

## Abstract

*Background and Objectives*: Anxiety is widely recognized as a common mental health issue. Extensive research highlights the benefits of adopting healthier lifestyle habits in improving both physical and mental wellbeing. This study aims to assess the levels of anxiety and physical activity, and if associations exist, among the population of Saudi Arabia. *Materials and Methods*: The study included a sample of 244 participants, who responded to an online survey containing demographic information, the Arabic versions of the General Anxiety Disorder-7 (GAD-7), and the Global Physical Activity Questionnaire (GPAQ) as assessment tools. *Results*: The study participants had a median age of 31 years and most of them were females (64.7%). Almost three quarters of the participants had received a higher education (73.5%) and were nonsmokers (94.1%). Assessing their physical activity levels, moderate or low-intensity physical activities, particularly walking and cycling, were common. Participants’ anxiety levels, measured by the GAD-7, were higher in females, students, and employees. Physical activity, especially walking or cycling, was linked to lower anxiety, while sedentary behavior, characterized by less than three hours of daily activity, was also associated with reduced anxiety. Interestingly, the duration of physical activity had no significant impact on anxiety levels. *Conclusions*: This study examined how lifestyle factors, including physical activity and sedentary behavior, influence anxiety levels among a cohort in Saudi Arabia. It emphasizes the need to encourage moderate-intensity activities and limit sedentary time, especially among high-risk groups, like students and women, to help alleviate anxiety.

## 1. Introduction

The World Health Organization (WHO) conceptualizes mental health as a “state of wellbeing in which the individual realizes his or her abilities, can cope with the everyday stresses of life, can work productively and fruitfully, and is able to contribute to his or her community” [1]. Given its vital role in the population’s wellbeing, it is essential to identify the factors that promote its preservation, as well as the factors that might influence it negatively.

Anxiety is commonly known as a mental health disorder. The WHO defines anxiety disorder as “excessive fear and worry and related behavioral disturbances”. Such a disorder is typically accompanied by physical tension and other behavioral and cognitive symptoms [2]. In Saudi Arabia, anxiety disorder has been described as being quite common, ranging between 12% and 65% [2,3,4] of the population, posing a significant public health concern. In attempts to tackle mental health disorders, numerous lifestyle factors were found to drive the morbidity and mortality associated with medical and psychiatric disorders, as well as their associated effects. These include, but are not limited to, the consumption of a poor diet, a sedentary lifestyle and lack of exercise, smoking, alcohol consumption, and abusing illegal drugs [5]. On the other hand, adhering to a healthy lifestyle enhances people’s mental health. A healthy lifestyle entails living in a healthier environment, obtaining enough sleep daily, having fun and stress-relieving hobbies, being part of supportive social networks, and having constructive mental pursuits [6]. While healthcare providers frequently use slogans like “no physical health without mental health” and “no mental health without physical health,” the majority of the public and many healthcare professionals are unaware of the significant body of research that explains how and why adopting healthier lifestyle factors has positive effects on both physical and mental health [7]. 

It is commonly known that engaging in frequent physical activity, i.e., activities that exceed the daily sedentary activities, reduces the risk of mental illness [8]. Given the wealth of evidence that supports the role of physical health in limiting mental health issues, the (WHO) recommends that adults engage in 150 min or more per week of moderate-intensity aerobic physical activity (MIPA), 75 min or more per week of vigorous-intensity aerobic physical activity (VIPA), or an equivalent combination of the two [9]. 

The WHO reports that Saudi Arabia has the highest rate of physical inactivity regionally, both among the Gulf Cooperation Council nations and in the Middle East. Furthermore, the eastern Mediterranean region has the highest frequency of physical inactivity, at 35% [10]. A recent study demonstrated that the percentage of people in Saudi Arabia who did not fulfill the WHO standards for physical activity ranged from 66.8% to 81.2%, regardless of their gender [11]. People with mental disorders are more likely to develop chronic physical illnesses such as heart disease, diabetes, arthritis, and asthma, which is consistent with the link between physical exercise and anxiety levels [12,13]. 

In response to the recent COVID-19 pandemic, Saudi Arabia was one of the countries that imposed a series of strict measures that limited people’s movement and activity levels [14]. These restrictions undoubtedly impacted the population’s mental health [15]. While there were previous studies that established the relationship between anxiety and physical activity pre-COVID-19 [16,17], such an association after the pandemic remains largely unexplored in Saudi Arabia.

### Study Aims and Objectives

This study aims to demonstrate anxiety levels and levels of physical activity among the population of Saudi Arabia. Such an aim entails assessing the levels of physical activity and anxiety among the population of Saudi Arabia, as well as the factors that may influence such levels.

## 2. Materials and Methods

### 2.1. Study Design and Participants

This is a cross-sectional study that allowed us to collect data from a large pool of subjects and compare differences between groups. The number of participants required for this study was estimated to be 1825 participants, based on a sample size calculation at a 95% confidence interval [18]. However, the response rate did not meet the required sample size. Despite all efforts to increase participation in this study, we could only obtain responses from 244 participants. A convenience random sampling was employed to recruit the study participants. Participants were recruited through social media and asked to complete the questionnaire. Informed written consent was obtained before answering the questionnaire. Inclusion criteria were adult participants aged 18 years and older who resided in Saudi Arabia at the time of the study and could read and understand Arabic. The exclusion criteria in this study were participants who were younger than 18 years old, those with physical disabilities, those who were bedridden, or those who had been diagnosed with psychiatric disorders. The study took place between April 2024 and January 2025. 

### 2.2. Study Tool

The study tool was formatted and distributed using the online questionnaire platform, Google Forms. The questionnaire was divided into three sections. The first section collected participants’ basic demographic information such as gender, age, and smoking status. The second section was the translated Arabic version of the Global Physical Activity Questionnaire (GPAQ) [19,20]. The GPAQ, developed by WHO, collected data on participants’ activity levels related to work, leisure, and transportation. Briefly, the GPAQ tool enquires about the frequency and duration of vigorous- and moderate-intensity activities as part of daily jobs or recreational activities. It also enquires about the frequency and duration of walking or using a bicycle as a means of transportation during the week.

The third section of the study tool contained the Arabic version of the General Anxiety Disorder-7 (GAD-7) [21,22], which was translated and validated to be used on a national level by the Saudi Ministry of Health. In short, the questionnaire is a 7-item tool with a 4-point frequency scale (Not at all, Several days, More than half of the days, Almost every day). Each response is given a score of 0, 1, 2, and 3, respectively. The total score indicates the participant’s overall anxiety levels as follows: 1–4 indicates minimal anxiety; 5–9 indicates mild anxiety; 10–14 indicates moderate anxiety; and 15–21 indicates severe.

### 2.3. Statistical Analysis

Descriptive statistics were used to describe the data. Frequencies and percentages were used to describe the categorical data. The Shapiro–Wilk test tested numerical data to assess the distribution of the data. Parametric (normally distributed) data were described using mean and standard deviations, while nonparametric data were described using median and interquartile ranges. 

Associations between categories in the data were analyzed using the chi-squared test. Comparisons between two parametric groups were conducted using Student’s t-test, while the Mann–Whitney U test was used to compare nonparametric data. One-way ANOVA was used to compare between more than two parametric groups, while nonparametric groups were compared using the Kruskal–Wallis test. Statistical significance was considered at a *p* value of less than 0.05. The statistical analysis was carried out using GraphPad Prism, version 10.2.

### 2.4. Data Management

The questionnaire results of each participant were stored in a password-protected folder. According to institutional regulations, multiple copies of this folder data were stored on a secure, cloud-based server that requires a username, password, and physical hard drives. Only the principal investigator and the research team had access to the raw data for this project’s duration. 

### 2.5. Ethical Considerations

The study participants were anonymized entirely, as no personal or identifiable information was collected about them. The study was conducted after obtaining ethical approval from the Taibah University College of Medicine Research Ethical Committee (STU-24-07) on 20 March 2024.

## 3. Results

### 3.1. Participants’ Characteristics

A total of 244 participants responded to the online questionnaire; however, 6 participants were excluded due to incomplete data. The remaining 238 participants completed the survey, of which 154 were female (64.7%); their median age was 31 years old. Over half of the participants were single ((n = 127) 53.7%) and the majority of participants (73.5%) received a high school education or higher (bachelor’s, master’s, or PhD). Almost half of the participants were employees (47.9%) and most of the participants (84%) were from the western region of Saudi Arabia. Detailed characteristics of the study participants are described in Table 1.

### 3.2. Participants’ Lifestyle

The majority of the study participants were nonsmokers (94.1%). The work of over 80% of the participants did not involve vigorous-intensity activity. In a typical week, over half of them had only two days or less of vigorous-intensity activities. One third of the participants spent 20–60 min participating in vigorous-intensity activities (39%). The work of half of the participants involved moderate-intensity activity (49.8%). The majority of the participants were involved in moderate-intensity activity for 3–5 days (55%). Half of the participants spent time participating in moderate-intensity activity for less than 20 min (48%). In a typical week, over 45% of the participants spent 2 or less days walking or cycling for at least 10 min continuously.

Half of the participants spent 20–60 min walking or bicycling for travel on a typical day (48.9%). Over half of the participants were not continuously engaged in vigorous-intensity sports for at least 10 min (56%) in a typical week and (45.7%) of participants spent 2 or less days engaging in vigorous-intensity sports. Nearly half of the participants (48.9%) spent 20–60 min engaging in vigorous-intensity sports on a typical day. Half of the participants were involved in moderate-intensity sports for at least 10 min continuously (50.4%). In a typical week, 45.5% of the participants spent 2 or less days engaging in moderate-intensity sports, and half of them spent around 20–60 min engaging in moderate-intensity sports on a typical day (51.17%).

Most of the participants spent 3–6 h sitting or reclining (46.8%) on a typical day. A detailed breakdown of the participants’ lifestyles and activities is described in (Table 2).

### 3.3. Determinants of Anxiety Among Participants

Participants’ anxiety levels were assessed and quantified using the GAD-7 score. The participants’ median GAD-7 score was 6, as mild anxiety was the most common among participants (41.6%), and severe anxiety was the least common (8.4%). A detailed analysis of participants’ anxiety levels, based on the GAD-7, is presented in Table 3.

**Figure 1:**
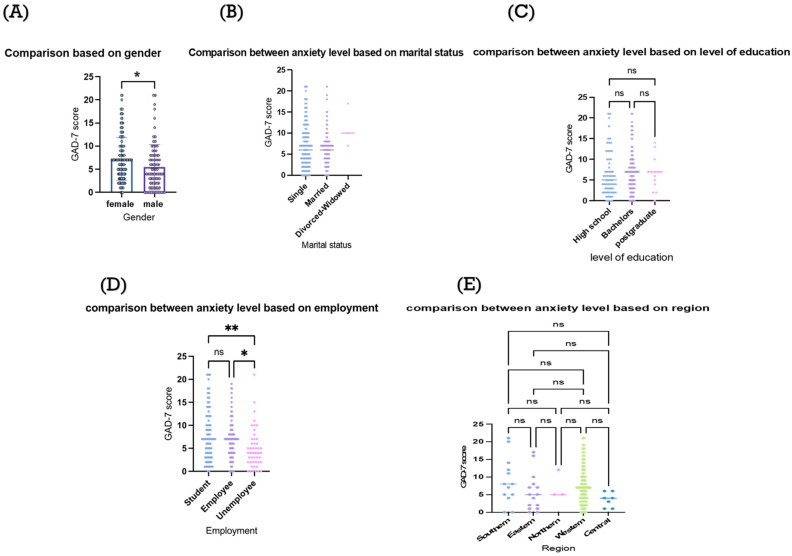
Comparisons of anxiety levels based on participants’ characteristics. The figure demonstrates comparisons of participants’ GAD-7 scores based on: (**A**) gender; (**B**) marital status; (**C**) level of education; (**D**) employment; and (**E**) region of residence. ns: statistically non-significant, * *p* value < 0.05, ** *p* value < 0.01. Mann–Whitney test (**A**) and Kruskal–Wallis tests (**B**–**E**) were used for comparison.

Female participants had significantly higher GAD-7 scores (*p*-value < 0.05) compared to male participants (Figure 1A). No significant difference was observed between single and married participants (*p*-value > 0.05) (Figure 1B). No significant differences (*p*-value > 0.05) were observed between participants based on their education levels (Figure 1C).

Students’ GAD-7 scores were significantly higher than those of non-employees (*p*-value < 0.01); employees had significantly higher GAD-7 scores than those of non-employees (*p*-value < 0.05; Figure 1D). No significant differences were observed between participants’ GAD-7 scores based on their place of residence (*p*-value > 0.05; Figure 1E) or smoking status.

### 3.4. The Association Between Physical Activities and Anxiety Levels

**Figure 2:**
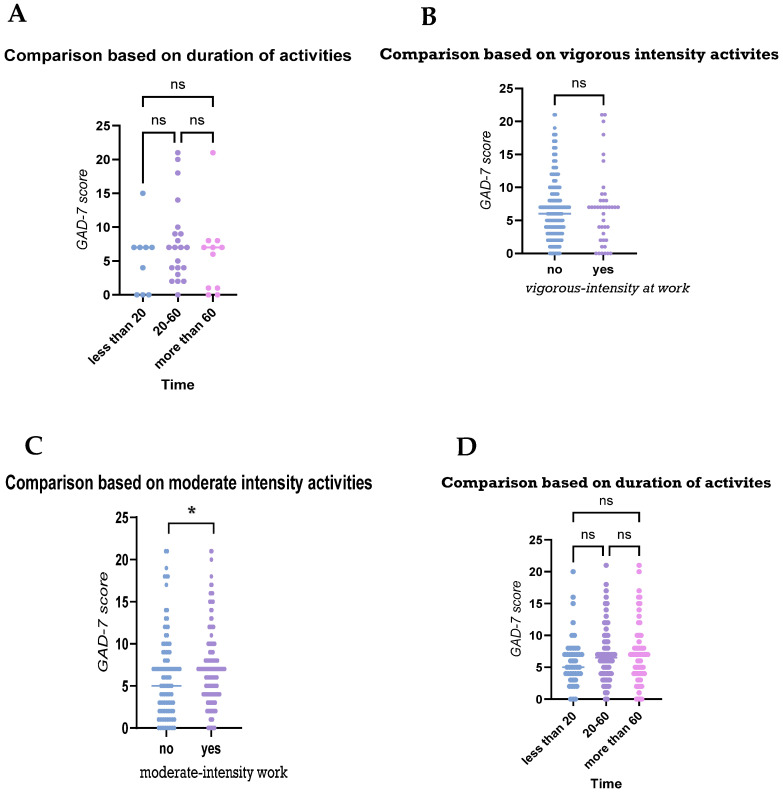
Comparisons of anxiety levels based on participants’ transportation physical activities. The figures demonstrate the levels between participants’ anxiety levels based on: (**A**) their engagement in physical activities, i.e., walking and/or cycling; and (**B**) the duration of that activity. ns: statistically non-significant, * *p* value < 0.05. Mann–Whitney test (**B**,**C**) and Kruskal–Wallis tests (**A**,**D**) were used for comparison.

Levels of anxiety among participants were compared based on their physical activities at work, during transportation, and recreational activities. Upon assessing anxiety levels between participants who were engaged in vigorously intense activities at work and those who were not, no statistically significant differences were observed (Figure 2A). Regarding the duration of the activity, the lack of statistically significant differences suggests that, in this sample, the amount of time spent in vigorous-intensity work does not appear to have a substantial impact on anxiety levels (Figure 2B). Upon assessing anxiety levels between participants who were engaged in moderate-intensity work, individuals who engaged in moderate-intensity work tended to have lower GAD-7 scores, suggesting a possible protective effect of physical activity on anxiety levels (Figure 2C). Similarly, the amount of time spent engaging in moderate-intensity work-related activities did not have a significant impact on participants’ GAD-7 scores (Figure 2D).

**Figure 3:**
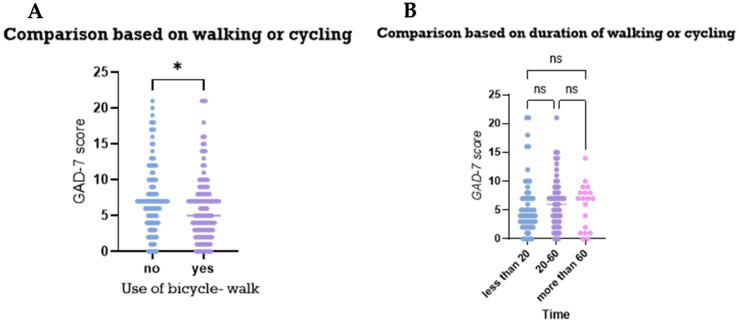
Comparisons of anxiety levels based on participants’ recreational physical activities. The figures display comparisons of the anxiety levels of the participants based on: (**A**) their engagement in vigorous activities and (**B**) their duration. ns: statistically non-significant, * *p* value < 0.05. Mann–Whitney test (**A**) and Kruskal–Wallis test (**B**) were used for comparison.

Upon assessing anxiety levels between participants based on their transportation activities, i.e., walking or cycling, those who used walking and/or cycling as transportation seemed to have lower anxiety levels compared to those who did not (*p* value < 0.05; Figure 3A). However, no differences were observed between participants based on the duration of their activities (*p* value > 0.05; Figure 3B).

**Figure 4:**
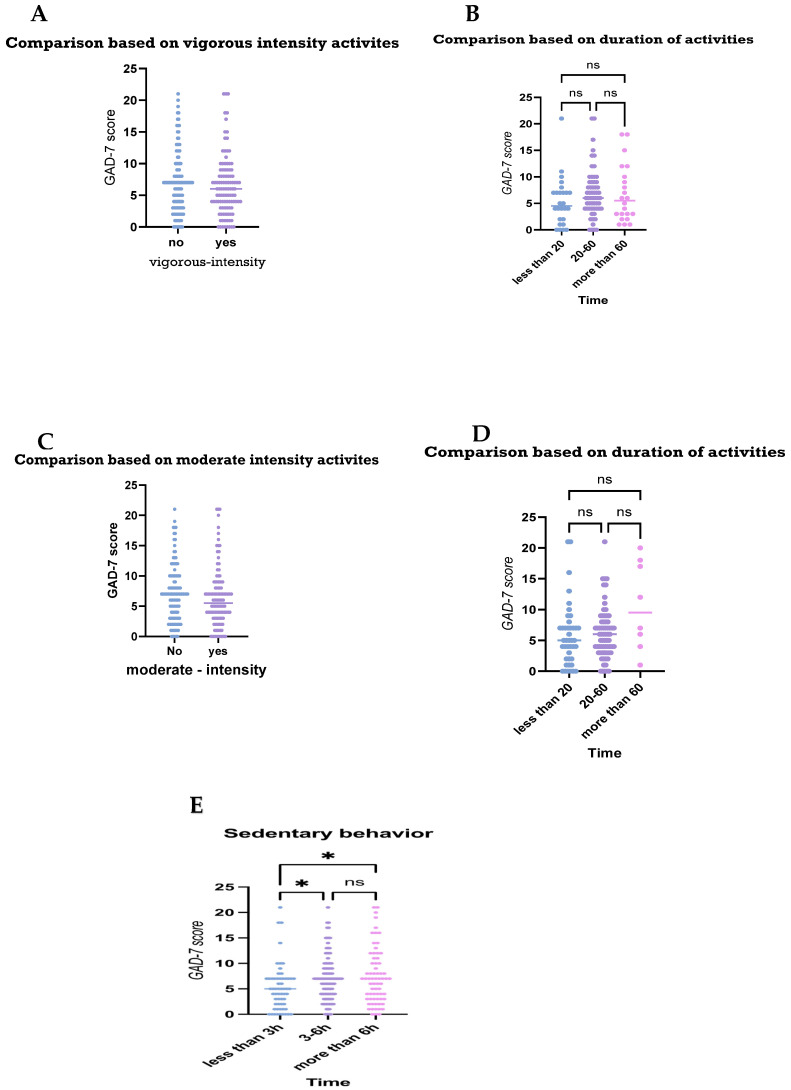
Comparisons of anxiety levels based on participants’ recreational physical activities. The figures display comparisons of the anxiety levels of the participants based on: (**A**) their engagement in vigorous activities; and (**B**) duration; (**C**) engagement in activities of moderate-intensity activities; (**D**) duration; and (**E**) the duration of their sedentary behavior. ns: statistically non-significant, * *p* value < 0.05. Mann–Whitney test (**A**,**C**) and Kruskal–Wallis tests (**B**,**D**,**E**) were used for comparison.

Upon assessing the anxiety levels of participants based on their physical activities, individuals who engaged in vigorous-intensity free-time activities tended to have moderately lower GAD-7 scores compared to those who did not, suggesting potentially lower anxiety levels in this group. However, there is a noticeable overlap in the ranges, suggesting that while vigorous-intensity activity might be associated with lower anxiety, there are still individuals with high GAD-7 scores in both groups (Figure 4A). However, the duration of the vigorous-activity free time did not significantly affect the anxiety levels measured by the GAD-7 score (Figure 4B).

Upon assessing anxiety levels between individuals who engaged in moderate-intensity recreational activities, no significant differences in the participants’ GAD-7 scores were observed in terms of their engagement in, or the duration of, the moderate recreational activities (*p*-value > 0.05; Figure 4C,D).

Interestingly, those who spent the least amount of time (less than 3 h per day) engaged in sedentary activities at home, i.e., sitting on the couch, had the lowest levels of anxiety, compared to those with who spent longer durations engaged in sedentary activities (*p* value < 0.05). However, there was no significant difference in anxiety levels between people who were sedentary for 3–6 h and those who were sedentary for more than 6 h (Figure 4E).

## 4. Discussion

This study aimed to evaluate anxiety levels, as indicated by GAD-7 scores, and physical activity among the population of Saudi Arabia, highlighting the various lifestyle and demographic factors that influence these aspects. The findings reveal important insights into the association between anxiety levels and physical activity in the Saudi population, as well as potential strategies for improving mental and physical wellbeing.

Our study results showed that female participants had significantly higher GAD-7 scores (*p*-value < 0.05) compared to male participants These results are in line with previous research that identified a similar relationship. This study contributes to the literature by confirming a higher risk of anxiety among females [23].

The impact of participants’ education levels on their anxiety levels was assessed as part of this study. Our results found no significant difference between postgraduates or those in high school or with a bachelor’s degree. Such findings are in contrast to other studies that showed that those with higher educational levels had significantly lower levels of anxiety [24]. The discrepancy between our results and the results of previously published studies could be explained by our study population, the majority of which were already educated at a university level (undergraduate or postgraduate), while those with lower levels of education represented just over one-fourth of the study population [25]. Furthermore, the association between education and anxiety levels is governed by gender [26,27], as the majority of participants in our study were females.

Additionally, it was reported that the age at which adults complete their high school impacted their anxiety levels [28]; however, this factor was not assessed in our study. Another study found a significant interaction between delayed high school completion and later educational attainment. The findings indicated that individuals who both experienced a delay in finishing high school and did not pursue higher education exhibited significantly higher levels of anxiety symptoms compared to all other groups based on high school completion timing and subsequent educational attainment [24]. Our study found no significant difference in anxiety levels between single and married participants, suggesting that relationship status alone may not significantly influence anxiety levels. In contrast, other research has shown that negative partner interactions are associated with higher levels of anxiety, depression, and suicidal ideation, while positive interactions are linked to better mental health [29].

This study demonstrated a significant inverse relation between anxiety and physical activity, but only for vigorous-intensity physical activity (not moderate-intensity physical activity or walking). Regarding the duration of activity, the lack of statistically significant differences suggests that in this sample, the amount of time spent in vigorous-intensity work does not appear to have a strong impact on anxiety levels. Upon assessing the anxiety levels between participants who are engaged in moderate-intensity work, individuals who engage in moderate-intensity work tended to have lower anxiety levels, suggesting a possible protective effect of physical activity on anxiety levels. Similarly, the amount of time spent in moderate-intensity work-related activities does not have a significant impact on participants’ anxiety levels [30].

This study possesses several strengths that validate its findings. First, the study tools, i.e., the questionnaires used in this study, are already established, reliable, and have been validated in previous studies. The use of validated questionnaires allows for the study to be replicated and for its findings to be generalized and used reliably in secondary analyses [31,32]. Another important positive aspect of the study is its timing. This study is among the first of its kind to evaluate the relationship between physical activity and anxiety levels after the COVID-19 pandemic. Previous research reported a significant rise in anxiety, with school-aged children experiencing rates between 1.8–23.87% and adolescents experiencing rates between 10.4–29.27%. In contrast, while anxiety remains prevalent, its patterns have shifted. Marital status showed no significant impact (*p* > 0.05). Postgraduates had lower anxiety scores than those with lower education levels, although this was not significant (*p* > 0.05), suggesting improved post-pandemic coping. Students reported the highest anxiety levels (*p* < 0.01), consistent with pandemic trends. However, employment-related stress has become a key factor, with employees showing higher anxiety than non-employed individuals (*p* < 0.05).

Compared to anxiety levels prior to the pandemic, the findings of this study demonstrate a higher prevalence of anxiety. Previously, reports suggested a prevalence ranging between 22% to 35% [33,34], whereas in our sample, the percentage of those with anxiety, regardless of its severity, exceeded 60%. Overall, post-pandemic anxiety is now driven more by work stress, financial stability, and regional disparities rather than health concerns and social isolation. These findings highlight the need for targeted mental health interventions for students and employees [15].

This study has several limitations that should be acknowledged. First, the cross-sectional nature of the study, and the lack of longitudinal data, limit the ability to infer causality between the variables assessed. Although this design is useful for identifying associations, it does not allow for the determination of temporal sequences, making it impossible to conclude whether exposure preceded the outcome or vice versa [35]. Consequently, any relationship observed should be interpreted with caution and viewed as correlational rather than causal.

Second, the sample size achieved fell short of the originally calculated requirement, which may have reduced the statistical power of the study. A smaller sample increases the risk of Type II errors—failing to detect significant effects where they exist—and may limit the ability to perform subgroup analyses or generalize the findings to the broader population [36]. Underpowered studies are a common limitation in observational research and highlight the need for careful recruitment planning and response-rate monitoring.

Third, the sample was predominantly composed of female participants. While this may reflect higher participation willingness among women in survey-based research, it introduces a potential gender bias. This is particularly relevant given that females have been consistently shown to report higher levels of anxiety and psychological distress compared to males in both clinical and non-clinical populations [37,38]. As such, the overrepresentation of females may skew the findings and limit the applicability of the results to male populations. Future studies should aim for more representative sampling across genders and consider using longitudinal or experimental designs to strengthen causal inferences and ensure broader generalizability.

## 5. Conclusions

This study highlights the significant role of lifestyle factors in shaping anxiety levels among the population of Saudi Arabia, pointing to the urgent need for targeted lifestyle interventions, especially after the COVID-19 pandemic. It stresses the necessity of minimizing sedentary behaviors and encouraging moderate-intensity physical activities that can be seamlessly incorporated into everyday life. Furthermore, the research reveals that demographic elements, including gender and academic-related stress, are crucial determinants of anxiety, as well as higher levels of anxiety prevalence, compared to the pre-COVID-19 era, indicating that public health strategies should prioritize the development of accessible and sustainable physical activity programs. Such initiatives should specifically aim to support vulnerable groups, particularly students and women, who exhibit a higher susceptibility to anxiety.

## Figures and Tables

**Table 1:** Participants’ characteristics.

Variable	Classification	Frequency (%)
Gender	Male	84 (35.3)
	Female	154 (64.7)
Age	Median (interquartile range)	31(22–40)
Marital status	Single	127 (53.7)
	Married	108 (45.4)
	Divorced	2 (0.84)
	Widowed	1 (0.42)
Educational level	High school	63 (26.5)
	Bachelor’s	161 (67.6)
	Master’s	10 (4.2)
	PhD	4 (1.7)
Employment	Student	77 (32.4)
	Employee	114 (47.9)
	Unemployed	47 (19.7)
Region of residence	Eastern	15 (6.3)
	Western	200 (84)
	Southern	13 (5.5)
	Northern	3 (1.3)
	Central	7 (3)

**Table 2:** Participants’ lifestyle and activity.

Variable	Classification	Frequency
Smoking	Smoker	14 (5.9)
	Non-smoker	224 (94.1)
Activity		
Work-related physical activities		
Work involving vigorous-intensity activity for at least 10 min continuously	Yes	40 (16.8)
	No	196 (82.4)
Number of days (per week) including vigorous-intensity activities as part of your work.	2 days or less	40 (50.6)
	3–5 days	30 (38)
	More than 5 days	9 (11.4)
Duration of the vigorous-intensity activities at work on a typical day.	Less than 20 min	28 (33.3)
	20–60 min	33 (39.3)
	More than 60 min	23 (27.3)
Work involving moderate-intensity activity for at least 10 min continuously.	Yes	118 (49.8)
	No	119 (50.2)
Number of days (per week) including moderate-intensity activities as part of your work.	Less than 2	53 (34)
	3–5	87 (55.8)
	More than5	16 (10.3)
Duration of the moderate-intensity activities at work on a typical day.	Less than 20 min	73 (48)
	20–60 min	54 (35.5)
	More than 60 min	25 (16.4)
Transportation physical activities		
Number of days (per week) involving walking or cycling for at least 10 min.	2 days or less	59 (45.7)
	3–5 days	56 (43.4)
	More than 5 days	14 (10.9)
Duration of walking or cycling for travel on a typical day.	Less than 20 min	43 (32.8)
	20–60 min	64 (48.9)
	More than 60 min	24 (18.3)
Recreational physical activities		
Doing vigorous-intensity sports for at least 10 min continuously.	Yes	104 (44.1)
	No	132 (56)
Number of days (per week) doing vigorous-intensity sports for at least 10 min continuously.	2 days or less	59 (45.7)
	3–5 days	56 (43.4)
	More than 5 days	14 (10.9)
Duration of doing vigorous-intensity sports on a typical day.	Less than 20 min	43 (32.8)
	20–60 min	64 (48.9)
	More than 60 min	24 (18.3)
Doing moderate-intensity sports for at least 10 min continuously.	Yes	119 (50.4)
	No	117 (49.6)
Number of days (per week) doing moderate-intensity sports for at least 10 min continuously.	2 days or less	66 (45.5)
	3–5 days	60 (41.4)
	More than 5 days	19 (13.1)
Duration of doing moderate-intensity sports on a typical day.	Less than 20 min	58 (40)
	20–60 min	75 (51.7)
	More than 60 min	12 (8.3)
Duration of sitting or reclining on a typical day.	Less than 3 h	59 (24.9)
	3–6 h	111 (46.8)
	More than 6 h	67 (28.3)

**Table 3:** GAD-7 among participants.

Anxiety Level (GAD-7 Score)	Number of Participants (%)
Minimal (0–4)	88 (16%)
Mild (5–9)	99 (41.6%)
Moderate (10–14)	31 (13%)
Severe (more than 15)	20 (8.4%)

## Data Availability

The original contributions presented in this study are included in the article. Further inquiries can be directed to the corresponding author.
